# Deer velvet powder-induced antral stricture mimicking infantile hypertrophic pyloric stenosis in a 3-month-old infant: a case report

**DOI:** 10.1308/rcsann.2025.0026

**Published:** 2025-04-24

**Authors:** CE Azmat, U Mahmood, N Talat, MB Mirza, WU Rehman, R Khalid

**Affiliations:** The Children Hospital and University of Child Health Sciences, Lahore, Pakistan

**Keywords:** Deer velvet powder, Infantile hypertrophic pyloric stenosis, Gastric outlet obstruction, Billroth I procedure

## Abstract

Antral stricture is a rare cause of gastric outlet obstruction in infants, typically linked to infantile hypertrophic pyloric stenosis (IHPS). We report a case of a 3-month-old male with a 1.5-month history of progressively worsening, nonbilious vomiting. Although initial imaging suggested IHPS, intraoperative findings revealed a sealed antral perforation with dense adhesions, leading to a distal gastrectomy with Billroth I reconstruction. A detailed retrospective history disclosed that the infant had been given deer velvet powder, suspected to have contributed to the perforation and subsequent stricture formation. This novel association highlights potential risks of unregulated supplement use in infants and underscores the need for further investigation.

## Background

Antral stricture is a rare cause of gastric outlet obstruction in neonates and infants, commonly associated with a history of corrosive substance ingestion in older children. Gastric outlet obstruction does not represent a singular condition; instead, it encompasses any pathology that results in mechanical impairment of gastric emptying. In infants, this condition is attributed predominantly to infantile hypertrophic pyloric stenosis (IHPS).^1^ Other causes include peptic ulcer disease, motility disorders and anatomical anomalies such as pyloric atresia, stenosis, web or diaphragm.^2^ Most IHPS cases present between three and ten weeks of life.^3^ Corrosive ingestion in paediatric populations, while uncommon in neonates, can also lead to gastric outlet obstruction, with peak incidence in children aged one to five years.^4^

We report a case of antral stricture in a 3-month-old infant, presenting with symptoms resembling IHPS. The condition was suspected to have been caused by deer velvet powder (DVP). DVP is a supplement made from the soft, developing antler tissue of deer before it hardens.^5^ It is dried, ground into a fine powder, and used commonly in traditional and alternative medicine for its potential health benefits.^6^ Deer velvet powder contains a variety of minerals, including calcium, phosphorus, sulphur, magnesium, potassium, sodium, manganese, zinc, copper, iron, selenium and cobalt, as well as amino acids and free fatty acids. Although deer antler is often promoted for its health and performance benefits, these claims are based primarily on anecdotal evidence rather than scientific research.^7^

## Case history

A 3-month-old male born at term via spontaneous vaginal delivery presented to the emergency department with a 1.5-month history of vomiting. Initially nonprojectile, the vomiting became projectile over time, remained nonbilious, and was associated with milk intake. There was no associated fever or diarrhoea.

On examination, the infant was malnourished, dehydrated and lethargic, with tachycardia. The weight was 3kg (<fifth percentile for age), and the abdominal examination was unremarkable, with no palpable masses. Initial management included resuscitation with intravenous fluids. Arterial blood gases revealed metabolic alkalosis, and baseline investigations were conducted. The child was admitted with a preliminary diagnosis of chronic gastroesophageal reflux disease (GERD).

The ultrasound demonstrated a pyloric canal length of 20mm, a pyloric diameter of 10mm and a wall thickness of 3.5mm. Based on these measurements, the pyloric canal length met the criteria for IHPS, although the pyloric diameter and wall thickness were borderline. Abnormal arterial blood gas values were also observed. The patient was transferred to the surgical unit, where IHPS management protocol was initiated. The patient started on intravenous fluids 1.5 times normal and in addition was given normal saline boluses (20ml/kg) to correct the electrolyte imbalance as per the bolus therapy. After optimisation, the child was scheduled for elective surgery.

**Figure 1:**
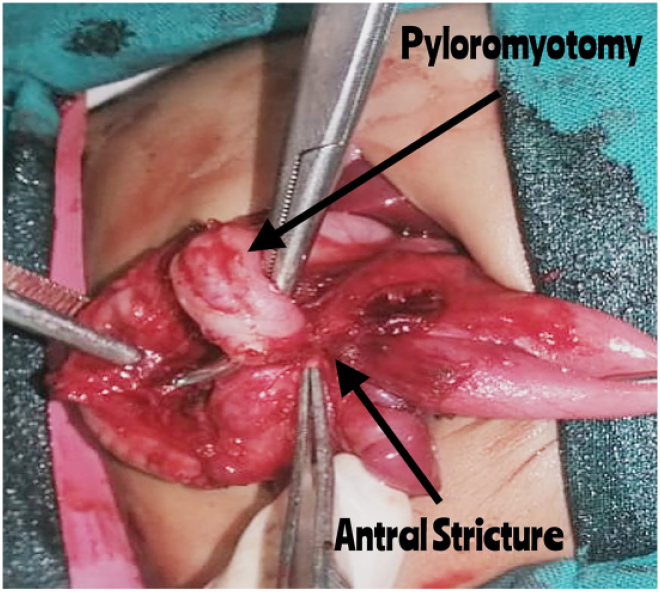
A picture showing the performed Pyloromyotomy and antral stricture densely adherent to transverse colon.

**Figure 2:**
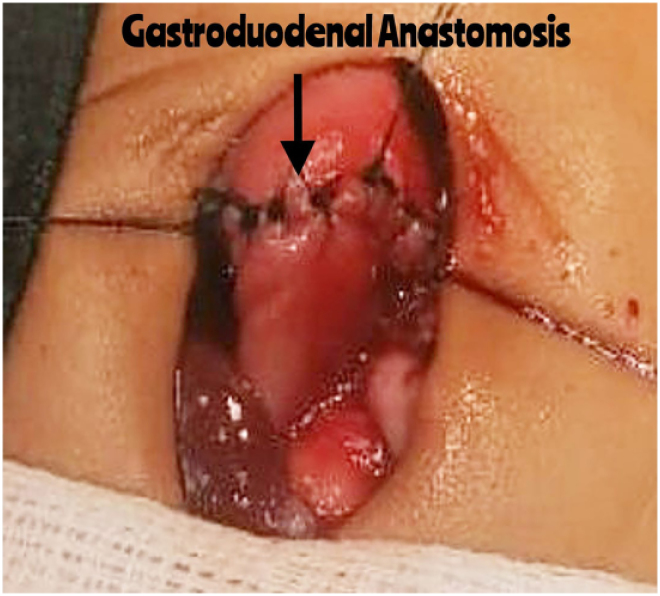
An intraoperative picture showing performed gastroduodenal anastomosis

During the operation, a standard right transverse laparotomy incision was used for exploration. Given the observation of a mildly thickened pylorus, pyloromyotomy was performed. During intraoperative assessment, while testing the stomach’s mucosal integrity, the initial incision was extended due to unexpected findings. A sealed perforation was discovered in the antrum, accompanied by dense adhesions involving the transverse colon, pancreas and spleen (Figure 1), along with a pinpoint opening in the antrum. Given the complexity of the antral stricture and its adherence to surrounding structures, the surgical team elected to perform a distal gastrectomy with a gastroduodenal anastomosis (Billroth I procedure) to ensure complete removal of compromised tissue and restoration of gastrointestinal continuity (Figure 2). A nasogastric tube was placed beyond the anastomosis, and the patient was transferred to the intensive care unit (ICU) for postoperative care.

Postoperative recovery was uneventful. On the seventh day, an oral contrast study confirmed repair integrity. Oral feeding was reintroduced gradually, and the patient tolerated feeds without vomiting. Postoperatively, further inquiry revealed that, following advice from elders, the parents had been administering DVP to the infant. A detailed retrospective history confirmed this intake, which likely precipitated an antral perforation that led to dense adhesions and subsequent stricture formation.

At discharge, the patient was able to feed orally and was passing stools normally. During follow-up visits, the patient had no complaints.

## Discussion

The incidence of gastric outlet obstruction in neonates and infants is poorly documented outside IHPS cases. A study in Taiwan identified non-IHPS causes in 11 out of 142 patients with gastric outlet obstruction.^8^ Corrosive ingestion in neonates and infants is exceedingly rare, with few cases reported. Hassan *et al* documented a rare case of corrosive ingestion in a 4-day-old newborn, where liquid bleach was accidentally mixed with formula milk. The infant initially presented with excessive crying, cyanosis and oral mucosal excoriation, later developing gastric perforation and pneumoperitoneum. Despite undergoing gastric repair and a feeding jejunostomy, the patient ultimately succumbed to sepsis postoperatively.^4^

Isa *et al* documented 17 cases of caustic ingestion in infants, making up 54.8% of all caustic cases, with vomiting and coughing as the most common symptoms. Management primarily involved endoscopy (58.1%) and omeprazole (67.9%), while lower socioeconomic status was associated with higher complication rates (*p*=0.028). Their findings highlight the importance of early diagnosis, endoscopic evaluation and caregiver education to prevent severe outcomes.^9^

Abdulkadir *et al* reported a rare case of accidental sulphuric acid ingestion in a 6-hour-old newborn, mistakenly given as holy water (Zam Zam). The infant developed haematemesis, respiratory distress, gastrointestinal bleeding and bowel perforation.^10^ However, no previous cases of DVP causing gastric injury have been reported.

Most studies on the effects and potential risks of DVP have been conducted on animals rather than humans.^11^ However, a review of the available literature reveals no documented cases of DVP causing gastrointestinal perforations or strictures. In fact, only one case of a delayed antral stricture, as a late complication due to iron sulphate tablet ingestion, has been documented, in Texas.^12^

In this case, the stasis of DVP in the stomach may have led to perforation, which then resulted in dense adhesions, ultimately leading to an irreparable distal stomach. A meta-analysis has shown that common substances causing gastric outlet obstruction due to intoxication include household cleaning agents, particularly bleaches and other cleaners.^13^ We believe this may represent a novel case of DVP causing an antral stricture followed by perforation.

## Conclusion

This case presents a novel association between DVP ingestion and antral stricture with perforation in an infant. Whereas gastric outlet obstruction in neonates and infants is uncommon beyond IHPS, this case raises concerns about the potential risks of dietary supplements in this vulnerable population. With no previous reports linking DVP to gastric perforation or stricture formation, these findings emphasise the need for further research and greater awareness regarding the dangers of unregulated supplement use in infants.

## Author Contribution

All authors attest that they meet the current ICMJE criteria for Authorship.

## Ethical Approval

Ethical approval was not required for this case report as per hospital guidelines, as it involves the description of a single clinical case and does not constitute systematic research. Written informed consent for publication of this report, including the use of clinical details and images, was obtained from the patient’s legal guardian.

## Consent

Informed consent was taken from the parents.

## References

[1] Sharma KK, Ranka P, Goyal P *et al.* Gastric outlet obstruction in children: an overview with report of “Jodhpur disease” and Sharma’s classification. *J Pediatr Surg* 2008; **43****:** 1891–1897. 18926227 10.1016/j.jpedsurg.2008.07.001

[2] Shukla RM, Mukhopadhyay M, Tripathy BB *et al.* Pyloric and antral strictures following corrosive acid ingestion: a report of four cases. *J Indian Assoc Pediatr Surg* 2010; **15****:** 108. 21124669 10.4103/0971-9261.71749 PMC2980922

[3] Lone YA, Hushain D, Chana RS *et al.* Primary acquired gastric outlet obstruction in children: a retrospective single center study. *J Pediatr Surg* 2019; **54****:** 2285–2290. 30922687 10.1016/j.jpedsurg.2019.02.056

[4] Hassan A, Munir A, Naumeri A *et al.* Corrosive ingestion in a 4-day-old newborn. *J Pediatr Adolesc Surg* 2022; **2****:** 39–40.

[5] Zhang R, Li Y, Xing X. Comparative antler proteome of sika deer from different developmental stages. *Sci Rep* 2021; **11****:** 10484. 34006919 10.1038/s41598-021-89829-6 PMC8131589

[6] Sui Z, Zhang L, Huo Y *et al.* Bioactive components of velvet antlers and their pharmacological properties. *J Pharm Biomed Anal* 2014; **87****:** 229–240. 24029381 10.1016/j.jpba.2013.07.044

[7] Sleivert G, Burke V, Palmer C *et al.* The effects of deer antler velvet extract or powder supplementation on aerobic power, erythropoiesis, and muscular strength and endurance characteristics. *Int J Sport Nutr Exerc Metab* 2003; **13****:** 251–265. 14669926 10.1123/ijsnem.13.3.251

[8] Otjen JP, Iyer RS, Phillips GS *et al.* Usual and unusual causes of pediatric gastric outlet obstruction. *Pediatr Radiol* 2012; **42****:** 728–737. 22457062 10.1007/s00247-012-2375-5

[9] Isa HM, Aldoseri SA, Abduljabbar AS *et al.* Accidental ingestion of foreign bodies/harmful materials in children from Bahrain: a retrospective cohort study. *World J Clin Pediatr* 2023; **12****:** 205–219. 37753493 10.5409/wjcp.v12.i4.205 PMC10518745

[10] Abdulkadir I, Hassan L, Abdullahi F *et al.* Accidental sulphuric acid poisoning in a newborn. *Niger J Paediatr* 1970; **42****:** 237–240.

[11] Park KH, Park CI, Seo JW *et al.* Anti-fatigue effect of an enzymatically derived deer velvet extract through muscle damage recovery and improvement of antioxidant levels. *J Ethnopharmacol* 2025; **337****:** 118965. 39427740 10.1016/j.jep.2024.118965

[12] Vuthibhagdee A, Harris NF. Antral stricture as a delayed complication of iron intoxication. *Radiology* 1972; **103****:** 163–164. 5015817 10.1148/103.1.163

[13] Rafeey M, Ghojazadeh M, Sheikhi S *et al.* Caustic ingestion in children: a systematic review and meta-analysis. *J Caring Sci* 2016; **5****:** 251–265. 27757390 10.15171/jcs.2016.027 PMC5045959

